# The concept of direct approach to lateral tibial plateau fractures and stepwise extension as needed

**DOI:** 10.1007/s00068-020-01422-0

**Published:** 2020-06-30

**Authors:** Karl-Heinz Frosch, Alexander Korthaus, Darius Thiesen, Jannik Frings, Matthias Krause

**Affiliations:** Department of Trauma and Orthopaedic Surgery, University Medical Center Hamburg-Eppendorf, Hamburg, Germany

**Keywords:** Tibial plateau fracture, Posterolateral corner, Surgical approach, Anatomic reduction, Osteosynthesis

## Abstract

Malreduction after tibial plateau fractures mainly occurs due to insufficient visualization of the articular surface. In 85% of all C-type fractures an involvement of the posterolateral-central segment is observed, which is the main region of malreduction. The choice of the approach is determined (1) by the articular area which needs to be visualized and (2) the positioning of the fixation material. For simple lateral plateau fractures without involvement of the posterolateral-central segment an anterolateral standard approach in supine position with a lateral plating is the treatment of choice in most cases. For complex fractures the surgeon has to consider, that the articular surface of the lateral plateau only can be completely visualized by extended approaches in supine, lateral and prone position. Anterolateral and lateral plating can also be performed in supine, lateral and prone position. A direct fixation of the posterolateral-central segment by a plate or a screw from posterior can be only achieved in prone or lateral position, not supine. The posterolateral approach includes the use of two windows for direct visualization of the fracture. If visualization is insufficient the approach can be extended by lateral epicondylar osteotomy which allows exposure of at least 83% of the lateral articular surface. Additional central subluxation of the lateral meniscus allows to expose almost 100% of the articular surface. The concept of stepwise extension of the approach is helpful and should be individually performed as needed to achieve anatomic reduction and stable fixation of tibial plateau fractures.

## The problem of malreduction after tibial plateau fractures

The clinical result after tibial plateau fractures depends on the quality of reduction. Multiple publications underlined this fact. Singleton et al. have shown that a postoperative step in the articular joint surface of less than 2.5 mm results in a significant better range of motion, lesser pain and an improved KOOS Score [1]. Similar results were published by Parkkinen et al. who demonstrated that a step of < 2.0 mm is associated with lesser development of osteoarthritis after 4 years [2]. Axis deviations in the frontal plane of less than 5° also had a significant lower rate of osteoarthritis of the knee [3]. The same group has also shown that steps of more than 2.0 mm and a varus deformity of more than 4° are risk factors for the development of osteoarthritis [2, 4]. These results indicate that anatomic reduction is a prerequisite for a good clinical outcome.

Risk factors for failure of primary surgery of complex tibial plateau fractures were inadequate experience of the surgeon, inaccurate diagnosis and management, improper selection of implants, and poor surgical techniques [5]. Instead, adequate preoperative evaluation, accurate intraoperative procedures, and proper postoperative rehabilitation are also prerequisites for a successful treatment in revision surgeries [5]. Meulenkamp et al. examined 65 patients with tibial plateau fractures after surgical treatment by computed tomography (CT) scan and observed a postoperative step of > 2 mm in 32.3% of all patients. Age, body mass index, OTA/AO fracture type, operative time, use of bone graft or bone graft substitute, and use of locking plates were not predictive of malreduction [6]. Malreductions were heavily weighted to the posterior quadrants of the lateral tibial plateau, especially in the posterolateral-central segment, which cannot be visualized by standard anterolateral approaches [6, 7]. According to the ten segment classification of the German Knee Society, in around 85% of all OTA/AO type C fractures the posterolateral-central segment is involved [8]. As this segment is very difficult to visualize, even by intraoperative fluoroscopy [7], it is also called the dark side of the knee [9] and is therefore one of the key determinants for the right choice of the approach [10, 11].

The lack of intraoperative visualization of the articular surface is also confirmed by other authors to be the main failure reason in complex tibial plateau fractures [12]. Therefore, especially in revision cases, extended approaches for an optimal exposure of the articular surface are recommended [12].

## Which approach for which fracture?

For a proper visualization of the articular surface, the right choice of surgical approaches is a key factor for anatomic reduction and stable fixation. Multiple surgical approaches for the treatment of tibial head fractures have been described. With an anterolateral standard approach only 36% of the articular surface of the lateral plateau can be exposed [10]. So mainly the anterolateral–lateral and the anterolateral-central segments as well as parts of the posterolateral–lateral segment can be exposed. Thus, an anterolateral approach is appropriate for anterolateral fractures. If the central area of the lateral plateau is involved, a complete visualization of the fractured articular surface is difficult, and cannot be reached by standard approaches in most cases. For a proper visualization of the central segments of the fracture, extended approaches are necessary [10, 13]. To extend the standard approaches, there are four main possibilities (Table 1).

**Table 1:** Comparison of extended lateral approaches to treat tibial plateau fractures

Extended approach	Advantages	Disadvantages
Anterolateral by Cho et al. (“posterolateral rim plating”)	Additional overview of central and posterior area	Irreversible damage of the LCL
Osteotomy of the lateral rim of the lateral tibial plateau	Improved visualization of articular surface compared to the standard anterolateral approach	Insufficient visualization (especially central segments) after reduction of the fracture and so high risk of postoperative malreduction
	Low risk to damage of the posterior neurovascular bundle	
Osteotomy of the fibula head	87% of the articular surface can be visualized	Extensile soft tissue damage
		Risk of peroneal nerve injury
		Risk of instability or ossification of the tibiofibular joint
		Compared to the lateral epicondyle osteotomy technically demanding and relative unstable fixation of fibula head (soft bone)
Osteotomy of the lateral epicondyle	Technical easier compared to fibular head osteotomy	Nonunion of the osteotomy is seldom but can lead to posterolateral rotational instability
	83% of the articular surface can be visualized	
	Low risk of peroneal nerve injury	
	Easy and stable refixation	

First, “Rim plating of posterolateral fracture fragments through a modified anterolateral approach in tibial plateau fractures” [14] is one possibility and makes a extensile release of the lateral collateral ligament (LCL) necessary. With a physiological opening of the lateral joint space of 2–4 mm and an additional retraction of the LCL with a Hohmann´s hook to get a sufficient exposure of the central and posterior segments, an irreversible damage of the LCL is unpreventable and cannot be recommended.

Second, an osteotomy of the lateral rim of the lateral tibial plateau is another possibility and can be used in specific cases to expose and reduce the central segments [15]. The disadvantage of this technique is, that a direct visualization of the articular surface after reduction of the fracture and closure of the osteotomy is insufficient in most cases. Therefore, there is still a high risk of postoperative malreduction.

The third possibility of an extension of the lateral approach is the osteotomy of the fibula head [16, 17]. With an osteotomy of the fibula head around 87% of the articular surface can be visualized [18]. Disadvantages of this technique is the extensile soft tissue damage around the fibula head, the necessity of exposure of the peroneal nerve with the risk of injury, disruption of the proximal tibiofibular syndesmosis with potentially resulting instability of the tibiofibular joint or ossifications around the joint, and the relative unstable osteosynthesis (compared to lateral epicondyle osteotomy) in the soft bone of the fibula head with tension of the biceps muscle and LCL almost perpendicular to the osteotomy plane [19].

**Fig. 1:**
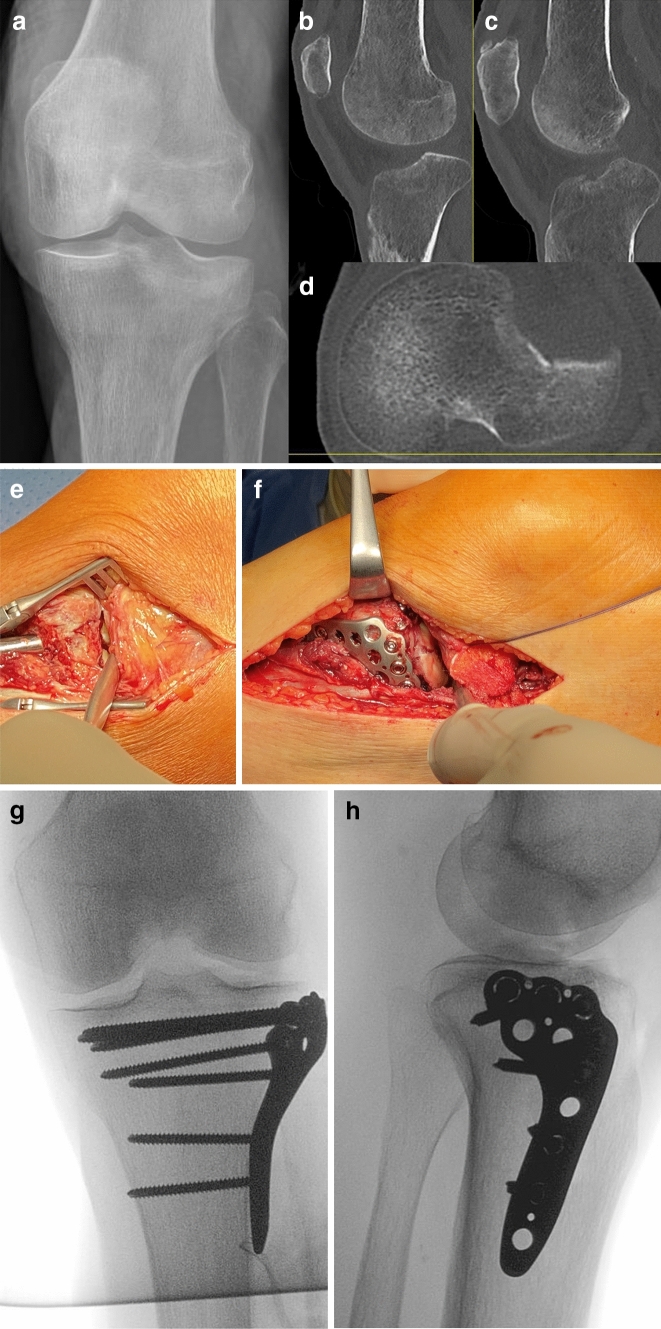
Lateral tibial plateau fracture with involvement of anterolatero–lateral and anterolatero-central (ALC) segments (**a**–**d**). The visualization of the ALC segment was not possible by standard anterolateral approach in this specific case (**e**). Extension of the approach by an osteotomy of the lateral epicondyle allowed an anatomic reduction under full visual control (**f**–**h**)

A technically easier procedure with a similar amount of exposure of the articular surface is the osteotomy of the lateral epicondyle, the fourth alternative [20, 21]. With this technique, around 83% of the articular surface can be exposed [18]. The advantage is that the peroneal nerve has not to be exposed, the procedure is easy and soft tissue preserving and the refixation with two lag screws is a safe and stable procedure [21]. We recommend to include both, the femoral footprint of the LCL as well as the popliteus tendon, in the osteotomy. The inclusion of the popliteus tendon in the osteotomy significantly improves the visualization of the lateral plateau and is therefore recommended by the authors [10, 18]. Usually the diameter of the osteotomy of the lateral epicondyle is 2 × 2 cm with at least 1 cm in depths (Fig. 1). To the experience of the authors with all 4 possibilities of extension of the lateral or anterolateral approach, the osteotomy of the lateral epicondyle is technically the easiest way with a sufficient area of visualization of the articular joint surface, with the lowest complication rates and the lowest extension of soft tissue damage. For the extension of the anterolateral or lateral standard approach to the lateral tibial plateau the osteotomy of the lateral epicondyle is therefore recommended [18]. This procedure can be performed in supine as well as in lateral or prone position [22].

## Arthroscopy and “fracturoscopy”

There are multiple publications about arthroscopic treatment of tibial plateau fractures. Especially AO type A, B1 and B2 can be indications for arthroscopic assisted treatment of tibial head fractures [23]. Concerning the quality of reduction it has been shown, that arthroscopic assisted treatment of tibial plateau fractures can be superior compared to open reduction [24]. But in this study mainly simple fracture patterns were included [24]. Even in posterolateral fractures arthroscopic reduction and percutaneous fixation can be successful [25]. For more complex intraarticular fractures, there is only one study up to now where the arthroscope was used to control fracture reduction, the so called “fracturoscopy” [7], which is an alternative for extended approaches concerning visualization of anatomic fracture reduction. “Fracturoscopy” describes the open insertion of the arthroscope during or after reduction of the fracture through the open surgical approach. By this procedure, the reduction of the articular surface, which is not fully visualized by the surgical approach, can be controlled with the arthroscope. It can be performed as a dry arthroscopy or with fluid rinsing out of the joint without producing any pressure in the soft tissue. It has been shown that especially in the involvement of the posterolateral-central segment, which cannot be visualized by the open standard lateral or anterolateral approach and also cannot be seen by fluoroscopic control in 8 out of 9 cases [7], “fracturoscopy” results in improved reduction of the articular joint surface. The indication for a “fracturoscopy” competes with an extended surgical approach. Up to now, there are no data preferring one or the other method. Compared to an extended approach with an osteotomy of the lateral epicondyle a “fracturoscopy” performed through a standard lateral or anterolateral approach is less invasive and a quick method to control fracture reduction. Especially in less complex fractures with involvement of the posterolateral-central segment the authors prefer a standard approach with “fracturoscopy”. In more complex fractures an extended approach is preferred. Especially when the surgeon decided to use a posterolateral buttress plate, an extended approach in lateral or prone position is favored and “fracturoscopy” redundant. Nevertheless the indication to use one or the other method depends on the experience and the skills of the surgeon. If the intraarticular fracture cannot be completely visualized either by fracturoscopy or by direct visualization, an additional intraoperative 3D imaging is strongly recommended. The authors strongly support that most intraarticular fractures of the lateral tibial plateau need to be visualized either by direct visualization through an open approach or by arthroscopy/fracturoscopy and additionally controlled by an intraoperative 3D scan. Otherwise steps or gaps of more than 2 mm will remain in at least 32% of the cases even if treatment was performed by experienced surgeons in a level 1 trauma center [6].

## Intraoperative 3D-imaging

Due to the anatomic structure of the tibial plateau in standard fluoroscopy parts of the tibial head are often overlaid by other bony structures. So an adequate depiction of the articular surface and fracture is not possible in fluoroscopy [26]. Intraoperative 3D scan has been comparable in most of the cases to postoperative CT scan in terms of quality of fracture reduction, with the added ability to intraoperatively evaluate implant positioning [27], joint width and geometry as well as small dislocated fragments in the intercondylar area. Beisemann et al. published an intraoperative revision rate of up to 32.4% with intraoperative 3D imaging after initial satisfying reduction under fluoroscopic control [28]. They also detected relevant intraarticular fragments, which would have remained undetected in conventional fluoroscopy [28]. Also, other authors have confirmed the benefit of intraoperative 3D imaging [29, 30]. Synoptically, in general the intraoperative 3D scan offers an advantage over conventional imaging.

Nevertheless, in complex tibial head fractures an intraoperative 3D imaging has a significant limitation, because it is a “retrospective” examination, which means that imaging or visualization occurs after reduction. Especially if the fracture is reduced and (temporarily) fixed without direct visual control, often steps and gaps in the articular surface remain. If a new reduction attempt is then performed again without direct visual control it can be again difficult to achieve a proper reduction requiring another 3D scan. To prevent multiple intraoperative 3D scans, the authors strongly recommend direct visualization or “fracturoscopy” during the process of fracture reduction. An intraoperative 3D scan is always a retrospective examination, which should be performed after reduction and (temporary) fixation of the fracture. It does not replace visualization during fracture reduction, which can only be performed by a proper and open approach (with or without extension) and eventually with additional fracturoscopy. Nevertheless, even after fragment reduction under direct visual control, the authors recommend an intraoperative 3D scan to judge positioning of osteosynthesis material, to rule out intraarticular screws and to secure that all key fragments are supported and fixed appropriately. Further dislocation of small fragments, closure of the rim, and the alignment of the complete lateral column can be controlled and documented.

## Stepwise extension of the surgical approach

**Fig. 2:**
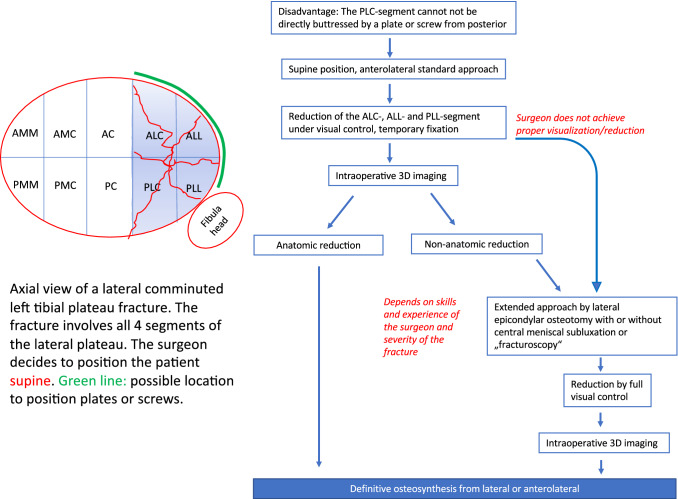
Algorithm of surgical care for lateral tibial plateau fractures in supine position

**Fig. 3:**
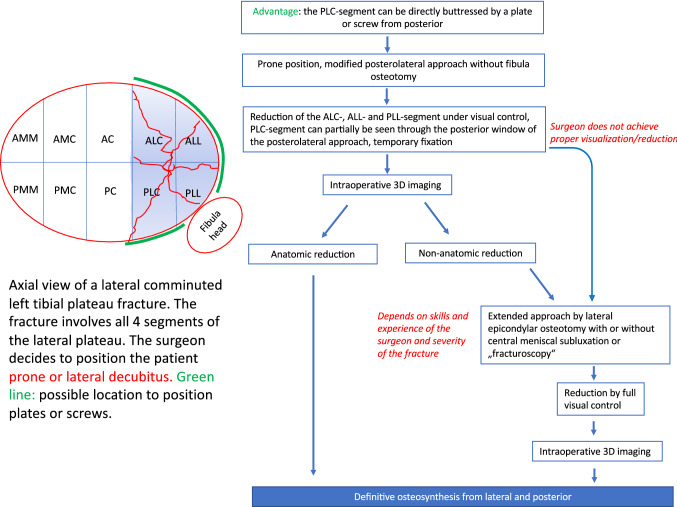
Algorithm of surgical care for lateral tibial plateau fractures in prone or lateral decubitus position

**Fig. 4:**
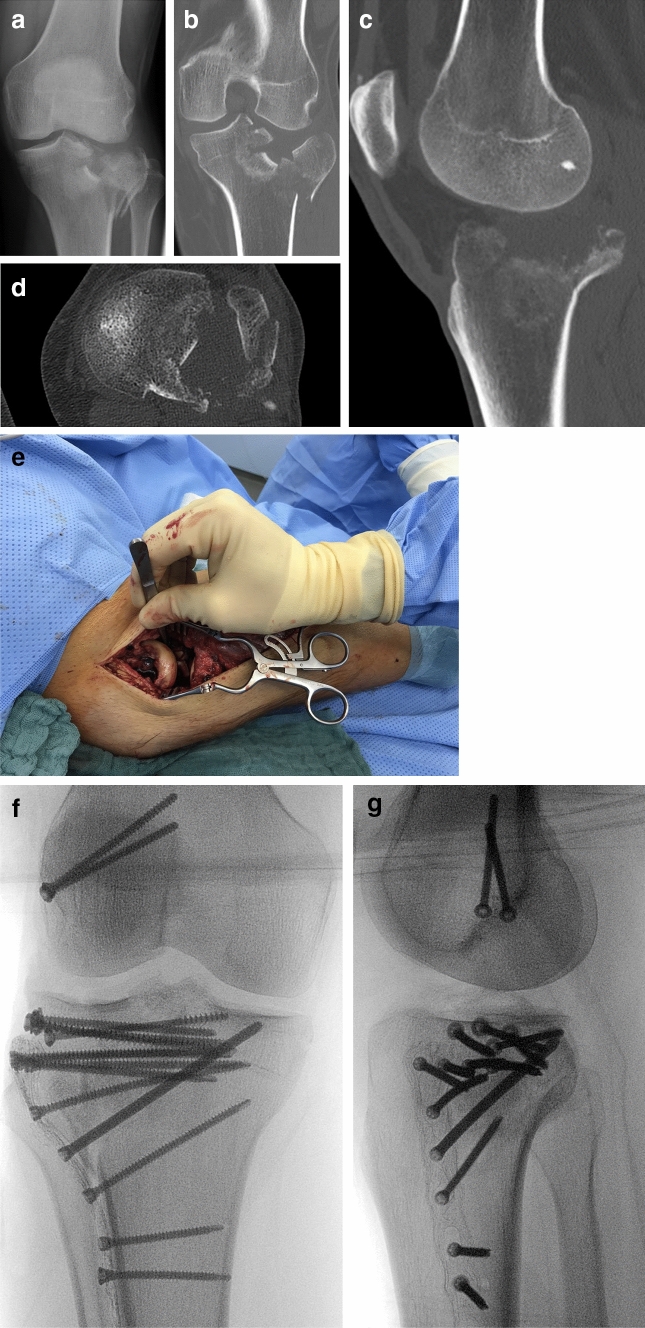
50 year old male with a comminuted lateral tibial plateau fracture (**a**–**d**). All segments of the lateral plateau are involved including both central segments (AC—anterior cruciate ligament, PC—posterior cruciate ligament). To reduce the fracture under full visual control including the whole lateral plateau and central segments, an extended approach with osteotomy of the lateral epicondyle [10, 12, 13] and a central subluxation of the lateral meniscus [31] were stepwise performed (**e**). With this treatment regimen an complete anatomic reduction of the articular surface was achieved (**f**, **g**). Fixation was performed by screws, lateral plate and a spongiosa allograft block underneath the articular surface after reduction of the fracture.

The goal of treatment of articular fractures in general is anatomic reduction of the articular surface and stable fixation. This goal was already declared by the pioneers of the AO in the 1970s and has continued to be a major goal in treatment of articular fractures up to now. To intraoperatively control the quality of fracture reduction there are four major tools like direct visualization, fracturoscopy, fluoroscopy and 3D imaging. Whatever tool the surgeon decides to use, he has to consider, that fluoroscopy is not a save procedure to control fracture reduction and 3D imaging is a “retrospective” examination and is recommended by the authors to use as final reduction control and documentation in complex articular fractures [7]. According to the literature, the current risk of malreduction in tibial plateau fractures is about 30–50%, if the fractured articular surface is not completely visualized. Most surgeons therefore prefer an open reduction. Hence, the choice of the surgical approach is of outstanding relevance [12]. Because extension of the approach is always an additional soft tissue trauma, the authors recommend a stepwise extension as necessary (Figs. 2, 3 and 4). The described concept is based on the ten segment classification of the German Knee society [8]. According to this classification system the surgeon can choose the segment, which he wants to visualize and buttress during surgery. For visualization of specific segments, specific approaches are necessary and were recently described [10–13]. With an anterolateral approach 36% of the articular surface of the lateral tibial plateau can be exposed [10]. With an extended anterolateral or lateral approach including a lateral epicondylar osteotomy more than 83% of the articular surface can be exposed. By an additional release of meniscocapsular fibers posterior to the popliteus tendon an central subluxation of the lateral meniscus can be performed and almost 100% of the articular surface of the lateral plateau can be visualized by an extended lateral approach and additional central meniscal subluxation [31]. The whole procedure can be performed in supine, prone or lateral position. But most fractures don´t need extended approaches. Especially most AO type A, B1 or B2 fractures can be treated by standard approaches without necessity of further extension. Therefore, extension of the surgical approach should only be performed, if it is required to expose additional areas of the articular surface, which helps the surgeon to control the fracture reduction under direct visual control. The concept of direct visualization of the intraarticular fracture is therefore a stepwise and individualized procedure depending on the fracture pattern, involvement on specific segments (especially posterolateral-central), experience and skills of the surgeon and individual factors of the patient.

## Limitations of the presented concept

Despite up to now there is no scientific proof that full visualization improves the clinical result, there are multiple papers indicating the failure of anatomic reduction mainly occurs due to insufficient visualization of especially the central segments. It is proven that these segments cannot be visualized by standard approaches but need extended approaches to expose articular surface. Even if the surgeon uses standard approaches for tibial head fractures he should be able to stepwise extend the approach as necessary.

## Fractures of the medial tibial plateau

“The Concept of Direct Approach to lateral Tibial Plateau Fractures and Stepwise Extension as needed” is also applicable for medial tibial plateau fractures. Because of different anatomy, different biomechanics and different fracture patterns there are also some differences in the treatment strategy of medial plateau fractures. Most fractures of the medial plateau are coronal split fractures, are associated in most cases with lateral plateau fractures and usually less complex than lateral plateau fractures [8]. With respect of anatomy and biomechanics the concept of treatment is comparable to the lateral side [13]. Nevertheless a detailed depiction of a differentiated concept of treatment of medial tibial plateau fractures would be worth to be thoroughly described in a separate article.

## Conclusion

The “concept of direct approach to lateral tibial plateau fractures and stepwise extension as needed” includes a complex algorithm based on the ten segment classification. Visualization and intraoperative control of fracture reduction is mandatory in surgical treatment of tibial plateau fractures. The concepts starts with the decision how the patient should be positioned during surgery. With a lateral or anterolateral standard approach only limited exposure of the articular surface is possible. To achieve anatomic reduction, it can be necessary to stepwise extend the surgical approach. Depending on the nature of the fracture, the extension of a lateral, anterolateral or posterolateral approach can be performed in supine, prone or lateral position. A stepwise extension with a lateral epicondylar osteotomy and a central subluxation of the lateral meniscus leads stepwise to an almost 100% exposure of the lateral articular surface [31]. An intraoperative 3D scan is a retrospective examination and should be performed in complex fractures after (!) fracture reduction and (temporary) fixation as additional control and documentation of anatomic reduction. A 3D scan has limited value as a reduction tool and cannot replace direct visualization of reduction. The concept of stepwise extension of the approach is helpful for proper visualization of the fractured articular surface, can prevent malreduction and should be individually performed as needed.
